# Reducing the Ideal Shear Strengths of ZrB_2_ by High Efficient Alloying Elements (Ag, Au, Pd and Pt)

**DOI:** 10.1038/srep43416

**Published:** 2017-02-24

**Authors:** Fu-Zhi Dai, Yanchun Zhou

**Affiliations:** 1 Science and Technology of Advanced Functional Composite Laboratory, Aerospace Research Institute of Materials & Processing Technology, Beijing 100076, China

## Abstract

Activating the plasticity of ZrB_2_ is a promising approach to improve its key properties for applications in hypersonic vehicles, including high temperature strength and thermal shock resistance. The present work demonstrates that ideal shear strength of ZrB_2_, which is a good indicator of the critical stress for dislocation nucleation, can be significantly reduced by dissolving of appropriate alloying elements. Analyzing on the bonding nature of ZrB_2_ reveals that choosing alloying elements with low energy valence electrons will prevent electron transferring from alloying element to the electron deficient B-B π orbits, which will reduce the local stability of the region surrounding the alloying element. Under the criterion, elements with *d* electrons tending to be full-filled (Ag, Au, Pd and Pt, the full-filled state is associated with low energy level) are selected as promising candidates with their prominent efficiency in reducing ideal shear strengths verified by first-principles calculations. The results provide useful guidelines for further designs of ZrB_2_ based materials, especially for improving their mechanical properties.

ZrB_2_ based ultra-high temperature ceramics (UHTCs) exhibit unique combination of excellent properties, including high melting point, chemical inertness, effective wear and environment resistance, which makes them promising for applications as shape leading-edges and nose-tips in hypersonic vehicles^1^,^2^. However, their practical uses at ultra-high temperatures are still impeded by the poor resistance to thermal shock and oxidation^2^ and the rapid drop of flexural strength at high temperatures^3^, e.g. the flexural strengths of ZrB_2_ based UHTCs usually drop down rapidly at temperature higher than 1200 °C^3^.

To realize the applications at evaluated temperatures, increasing attentions have been paid to the high temperature fracture strengths of ZrB_2_ based UHTCs^3^,^4^,^5^,^6^,^7^,^8^. A recent study by Wu *et al*.^4^ demonstrated that the retention of strength at evaluated temperatures is accompanied with substantial activation of dislocation plasticity in ZrB_2_ grains. The plastic process releases stress concentrations and dissipates substantial deformation energy, which reduces the possibility of sudden failure and results in a high fractural strength, which may in turn improve thermal shock resistance of ZrB_2_ based UHTCs. The plasticity mechanism is consistent with the fact that addition of TaSi_2_, MoSi_2_, WSi_2_ or WC generally improves the evaluated temperature strength of ZrB_2_^3^. In these materials, ZrB_2_ grains exhibit a core-shell structure with the shell dissolution of Ta, Mo or W^3^,^5^,^6^,^7^,^8^. Dislocations resulting from misfit or thermal-stress are found concentrated in the shell^5^,^6^,^7^, which indicates that dislocation nucleation in the shell become easier by dissolving these elements. These facts enlighten us that promoting the plasticity of ZrB_2_ by alloying may be a feasible way to improve the high temperature performance of ZrB_2_ based UHTCs. Therefore, the present work aims to search for high efficient alloying elements that can improve the plasticity of ZrB_2_.

To characterize the effects of alloy elements on the plasticity of ZrB_2_, the ideal shear strengths of ZrB_2_ with and without adding alloying elements were simulated by first-principles. Ideal strength can be defined as the stress necessary to induce permanent deformation (e.g. dislocations or cracks) in a material without prior imperfections^9^,^10^, which is an important material characterization. It implies that the ideal shear strength can be taken as an indicator on the critical stress for dislocation nucleation, which has been broadly adopted in investigations^11^,^12^,^13^,^14^. For example, the relative strength of Al and Cu can be well explained by the higher ideal shear strength of Al, even though Al displays a lower shear modulus^11^; the dominating dislocations activated under indentation in TMB_2_s (TM = Ti, Zr, Hf) can be indicated by the shear model with lower ideal shear strength^13^; the promoted dislocation nucleation in ZrB_2_ by alloying has been confirmed by the lower ideal shear strengths of TMB_2_s (TM = Nb, Ta, Mo, W) in comparison with those of ZrB_2_^14^, which well explained the concentrated dislocations in the shell. Therefore, evaluating the effect of alloying elements on the ideal shear strengths of ZrB_2_ is a promising guideline on the selection of elements that can improve the plasticity of ZrB_2_, since dislocation initiation is difficult for intrinsic brittle ceramics.

While it is difficult to acquire ideal strength from experiments, first-principles methods based on density functional theory provide a good choice to evaluate the ideal strength of materials^11^,^12^,^13^,^14^. In this wok, first-principles calculations were performed using the CASTEP code^15^ with the Vanderbilt-type ultrasoft pseudopotential^16^ and exchange-correlation described by generalized gradient approximation^17^. The plane wave cutoff energy was set to be 400 eV and *k*-points mesh with a separation of 0.04 Å^−1^ according to Monkhorst-Pack method^18^ was adopted in the Brillouin zone. 3 × 3 × 3 ZrB_2_ supercell with one Zr substituted by an alloying element was employed to model the solid solution, which means 3.7 at% of alloying element, as shown in Fig. 1. The selection of the supercell size is a tradeoff between computational cost and minimization of elastic interaction between alloying element and its image. The influence of elastic interaction to the substitution energy of alloying element is negligibly small at this supercell size. All the supercells were first optimized under zero pressure by using the Broyden-Fletcher-Goldfarb-Shanno (BFGS)^19^ minimization scheme. The convergence criteria for optimizations were set as follows: the difference in total energy within 1 × 10^−6^ eV/atom, the maximum ionic Hellmann-Feynman force within 0.002 eV/Å, the maximum ionic displacement within 1 × 10^−4^ Å, and maximum stress difference within 0.02 GPa.

To simulate the stress-strain relationship, the supercell was incrementally deformed in the imposed strain direction. At each step, the shear stress was increased by a value defined as 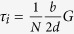, where *b* is the length of the Burgers vector, *d* is the interplanar spacing between the shear planes, *G* is the shear modulus, *N* is a number. In principle, a crystal recovers under a shear with magnitude of *b*/*d*. Therefore, *b*/2*d* can be taken as a rough upper limit of shear deformation because shear in the inverse direction usually results in a same stress-strain curve. *N* is selected to be 100, which results in *τ*_*i*_ around 2.5 GPa in the simulations. Under the applied stress, the crystal structure was fully relaxed until all other stress components vanished by using BFGS minimization with the convergence criteria as above. The relaxed cell was then taken as the starting structure for the next step to ensure the stress-strain curves being continuous. When approaching the ideal strength, the supercell is close to the instable point and the minimization algorithm is hard to converge. Two specific technologies were employed to guarantee that the calculated ideal strength was accurate enough. Firstly, the incremental stress was set to be one fifth of *τ*_*i*_. Secondly, if convergence could not be reached after adding the incremental stress, the incremental stress would be recursively reduced by half until convergence was reached. By doing so, the accuracy of the calculated ideal strength is around 0.2 GPa.

Experiments have revealed that dislocations activated under deformation in ZrB_2_ are dominated by <
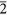
110>/3 dislocations^4^,^20^,^21^, where <
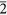
110>/3 is the Burgers vector. <
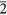
110>/3 dislocations slip on either {0001} plane or {01
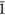
0} plane. Simulations on responds of chemical bonds to shear deformations demonstrate that the basal plane shear (shear in (0001) plane along [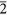
110]/3 direction) is governed by the strength of Zr-B bond, while the prismatic plane shear (shear in (010) plane along [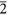
110]/3 direction) is controlled by both Zr-B bond and B-B bond^14^. Therefore, dissolution of alloying element X with weak X-B bond may reduce the barrier for dislocation nucleation, especially for the basal plane dislocations.

Instead of a thorough searching for all elements, the selection of X is guided by analyzing the chemical bonding nature in ZrB_2_. Typical chemical bonds in ZrB_2_ include B-B *σ* bond, B-B π bond and ionic-covalent bond between B and Zr^22^,^23^,^24^ (see the supplementary material for details), which is well represented by electron density difference map on the (11
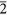
0) plane, as shown in Fig. 2a. Figure 2b illustrates electron density of state (DOS) of B and Zr. The DOS reveals strong coupling between valence electrons (4*d* electrons) of Zr and those (2*p* electrons) of B at an energy level above −4 eV, which indicates that the strong Zr-B bond is mainly originating from the coupling of B-B π bonds and Zr (see the supplementary material for details). Here, −4 eV is a rough boundary that separates energy level between B-B *σ* bond (formed by B-*sp*^2^ orbits) and B-B π bond (formed by B-*p* orbits)^24^ (see the supplementary material for details). Since valence electrons of B are only sufficient to fill the B-B *σ* bonds leaving the B-B π bonds empty, the electron deficient B-B π bonds need extra electrons transferred from the metal atoms, on average two extra electrons per metal atom. If B-B π bonds cannot capture sufficient electrons from metal atoms, the resultant bonding between the metal atom and B may be week in nature. It enlightens us that if the energy of valence electrons of the alloying elements is lower than that of B-B π bond, then transferring electrons from the alloying element to B will be energetically unfavorable. At this circumstance, no strong chemical bond will form between X and B. In principle, an element with high electronegativity value exhibits strong bind on its valence electrons, especially when valence electron orbits tend to be half-filled or full-filled. Then, the alloying elements can be chosen among those transition metals with high electronegativity values and *d* orbits close to half-filled or full-filled state. The criteria are consistent with the fact that Mo and W exhibit high efficiency in improving the plasticity of ZrB_2_, since they have large electronegativity values and their *d* electrons tend to be half-filled. The half-filled state reduces the tendency to bond with B and results in a weak Mo-B or W-B bond. In analogous, if the *d* electrons of X tend to be full-filled, then it is expected to be more efficient in improving the plasticity of ZrB_2_. Therefore, according to electronegativity values and valence states of elements, X is selected as Ag, Au, Pd or Pt (Table 1). After dissolving into ZrB_2_, the charges of these atoms analyzed by Mulliken population analysis^25^ are listed in Table 1. Comparing to the charge of Zr, all these elements exhibit much lower positive charge, which indicates unwillingness of charge transfer. The low charge values are coincident with the design criteria, which might be beneficial to reducing the bonding strength between X and B.

The difference of bonding between X-B and Zr-B is demonstrated by the electron density difference map on the (11
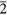
0) plane, as shown in Fig. 3a–d with X being Ag, Au, Pd or Pt, respectively. It is clear that the characteristics of X-B bonding are similar for these elements, which are different evidently from the Zr-B bonding. Due to the formation of strong chemical bonds, electron density between Zr and B exhibits substantial redistribution with strong enrichment of electron density between Zr and B. In contrast, charge redistribution between X (X = Ag, Au, Pd or Pt) and B is not as significant as that between Zr and B, particularly there is no evident positive redistributed electron density between X and B, which indicates a weak X-B bond. The weak bonding state between X and B can be explained by the DOS of B and X, as illustrated in Fig. 3e. It can be obtained from Fig. 3e that the dominating *d* electron peak of X deviates significantly from the *p* electron peaks of B, which means the *d* electrons of X tend to keep its own state instead of forming bond with B. In addition, the energy levels of *d* electrons of X are lower than those of B-B π bonds, e.g. the peak of *d* electrons of Ag and Au is located at −5 eV. It means that transferring these *d* electrons into B-B π bonds will increase the energy of the system, which is consistent with the objective of alloying element selection.

To assess the efficiencies of the selected elements, stress-strain curves of (Zr, X)B_2_ solid solutions for both basal plane shear and prismatic plane shear are simulated and compared with pure ZrB_2_ and (Zr, W)B_2_ solid solution, as shown in Fig. 4. Shear moduli (*G*, the slope of the linear part) and ideal shear strengths (*τ*, the maximum stress) determined from these curves are summarized in Table 1. The change in *G* and *τ* due to alloying is respectively evaluated by *G*/*G*_0_−1 and *τ*/*τ*_0_−1, where *G*_0_ and *τ*_0_ are shear modulus and ideal shear strength of ZrB_2_. Currently, W is known as the most efficient alloying element in reducing the ideal shear strength in ZrB_2_^14^, which make it a good reference to check the efficiency of other elements. As shown in Fig. 4, stress-strain curves with solid solution of W almost overlap with those of ZrB_2_, which shows that solid solution of W only displays softening effect at a strain exceeding 15% and finally reduces the ideal shear strength by 5.0% for the basal plane shear and 2.7% for the prismatic plane shear (Table 1). In contrast, the selected elements manifest more remarkable softening effect, where the stress-strain curves deviate from those of ZrB_2_ at small strains. The effects of these elements to the prismatic plane shear are moderate with the reduction in ideal strength being about 5.4%, 7.8%, 5.9% and 11.3% with respect to X being Ag, Au, Pd and Pt, which is more efficient than that of W. For the basal plane shear, the effect of solid solution is more substantial, the magnitude of reduction in shear modulus is around 10%, while the reduction in ideal strength is roughly twice that in shear modulus (Table 1). As has been mentioned previously, the basal plane shear is solely dominated by deformation of M-B (M represents metal) bond, while both M-B bond and B-B bond play key roles during the prismatic plane shear^14^. It explains why the impact to the basal plane shear is more remarkable. In addition, Table 1 still reveals that the softening effect of Ag, Au, Pd and Pt follows Au > Ag and Pt > Pd, which might result from the higher electronegativity of Au and Pt.

Due to the weak bonding nature of X-B bonds, the alloying elements will serve as seeds for dislocation nucleation. This effect can be verified by preferential elongation of chemical bonds surrounding X. Figure 5a illustrates the elongation-strain curves of X-B bonds and Zr-B bonds during the basal plane shear, while Fig. 5b demonstrates the elongation-strain curves of B-B_X_ bonds and B-B_Zr_ bonds during the prismatic plane shear. Here, B-B_X_ bond means the B-B bond surrounding X element. In Fig. 5, elongation-strain curves of Zr-B bond and B-B_Zr_ bond obtained from different simulations are almost coincident with each other. Therefore, they are not distinguished by different symbols. Figure 5a shows that elongation of any X-B bond is significantly higher than that of Zr-B bond, and the elongation differences exhibit an accelerating increase with the strain during the basal plane shear. Similarly, elongation of B-B bond surrounding the solute atom X is higher than that of B-B bond surrounding the Zr atom during the prismatic plane shear, as shown in Fig. 5b. Therefore, not only the bonding strength between X and B reduces by introducing of X, but also the strength of local B-B bond decreases. The weakened chemical bonds surrounding X element guarantee that deformation tends to localized and concentrates around the alloying element, which facilitates the local crystal to reach instability and generate a dislocation loop. In another word, the alloying element serves as the seed for dislocation nucleation.

For intrinsic brittle ceramics, activating their plasticity is a promising approach to improve their mechanical properties. When plastic process is efficiently activated (plasticity of ceramics can usually be activated at high temperatures), local stress concentrations can be released by dislocation motion, which will increase damage tolerance and strength of ceramics. Based on the idea of introducing weak bonds into crystals by alloying, the present work focuses on searching for high efficient alloying elements to reduce the critical stress for dislocation nucleation in ZrB_2_. The appropriate alloying elements are suggested to have high electronegativity value with their *d* orbits close to half-filled or full-filled state. Constrained by the criteria, alloying elements with *d* electrons tending to be full-filled (Ag, Au, Pd and Pt) were selected as promising candidates with their prominent efficiency verified by first-principles simulations. It does not mean that only alloying elements meet these two criteria can reduce the ideal shear strengths of ZrB_2_. In ZrB_2_, strong bonding between Zr and B is resulted from strong coupling between B-B π bond and 4*d* electrons of Zr, which needs transfer of electrons from Zr to B in energy range from −4 eV to 0 eV (the Fermi level). If an element dissolved into ZrB_2_ with energy levels of valence electrons lower than the energy levels of B-B π bond, transferring electrons from the element to B-B π bond is energetically unfavorable. Under these circumstances, the bonding between the alloying element and B will be weak in nature. In addition to the weak bonding between the alloying element and B, the strength of B-B bond surrounding the alloying element may also be reduced, such that the region around the alloying element will serve as a seed for dislocation nucleation. Introducing these elements into ZrB_2_ based UHTCs is expected beneficial to high temperature strength and toughness and in turn thermal shock resistance, which are main barriers for the practical applications of ZrB_2_ based UHTCs in hypersonic vehicles. In principle, the effect of alloying is comprehensive. Whether the alloying improves or deteriorates the overall performance of ZrB_2_ based UHTCs depends on the service environment. Nevertheless, fundamental knowledge of alloying effects on individual property is essential to the design of materials and analysis on their performance or failure.

## Additional Information

**How to cite this article:** Dai, F.-Z. and Zhou, Y. Reducing the Ideal Shear Strengths of ZrB**_2_** by High Efficient Alloying Elements (Ag, Au, Pd and Pt). *Sci. Rep.* **7**, 43416; doi: 10.1038/srep43416 (2017).

**Publisher's note:** Springer Nature remains neutral with regard to jurisdictional claims in published maps and institutional affiliations.

## Supplementary Material

Supplementary Information

## Figures and Tables

**Figure 1:**
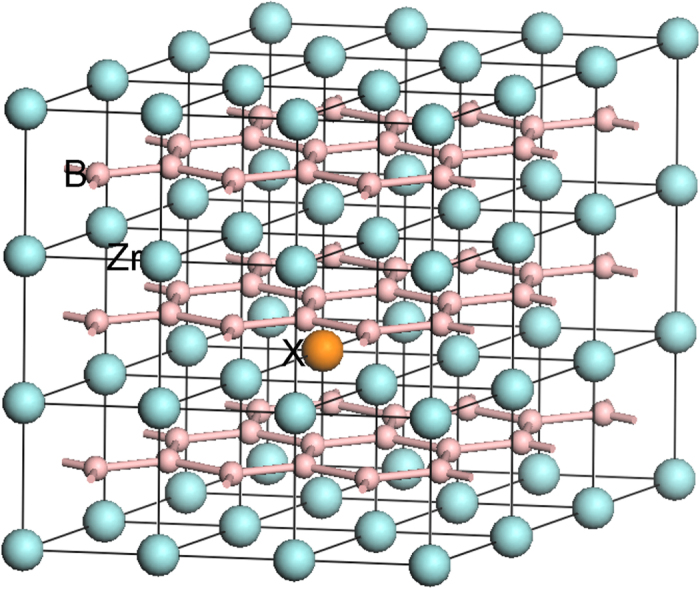
Illustration of a 3 × 3 × 3 ZrB_2_ supercell model with one Zr substituted by element X.

**Figure 2:**
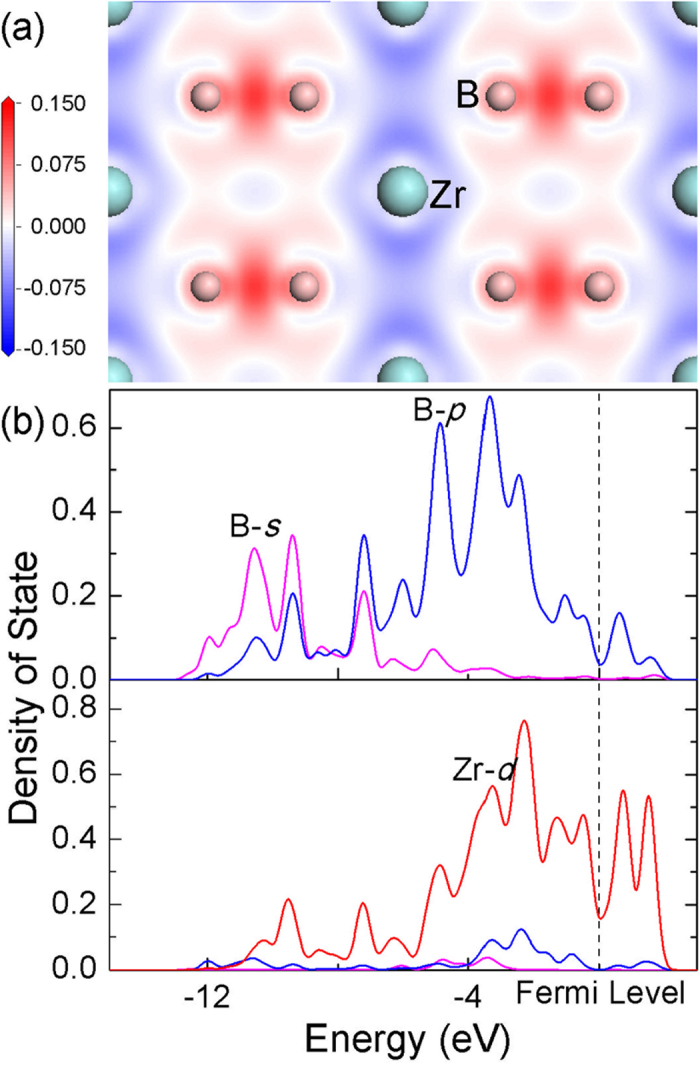
(**a**) Electron density difference map on (11
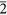
0) plane of ZrB_2_. (**b**) Density of state of B and Zr atom. Zero energy represents the Fermi level.

**Figure 3:**
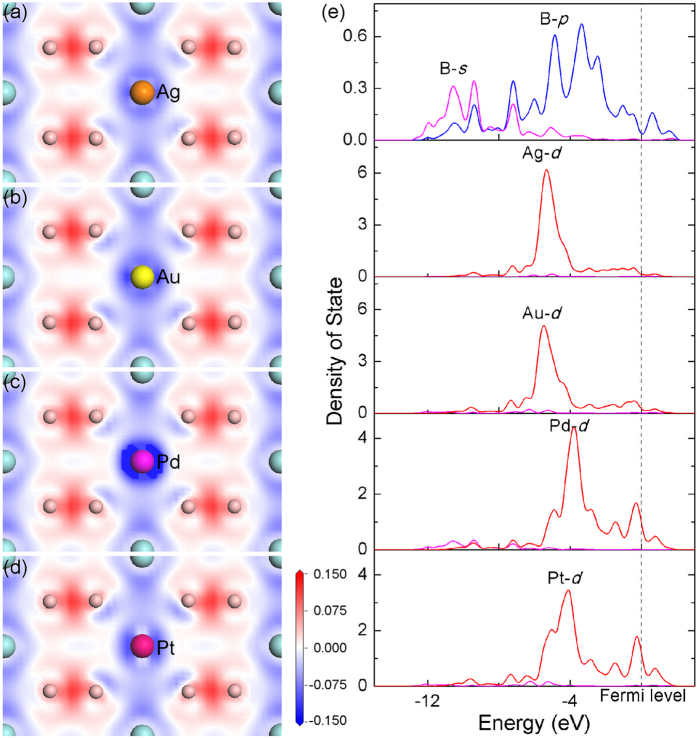
Electron density difference map on (11
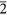
0) plane that contains X atom: (**a**) X = Ag, (**b**) X = Au, (**c**) X = Pd, (**d**) X = Pt. (**e**) Density of state of B and X atom. Zero energy represents the Fermi level.

**Figure 4:**
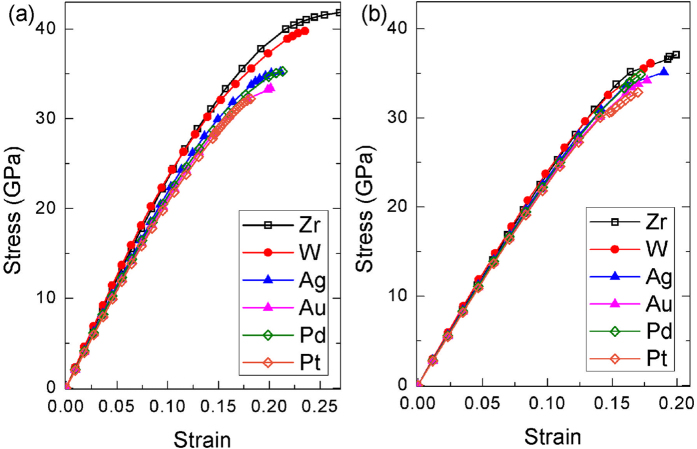
Simulated stress-strain curves for ZrB_2_ with different solute atoms: (**a**) the basal plane shear, (**b**) the prismatic plane shear.

**Figure 5:**
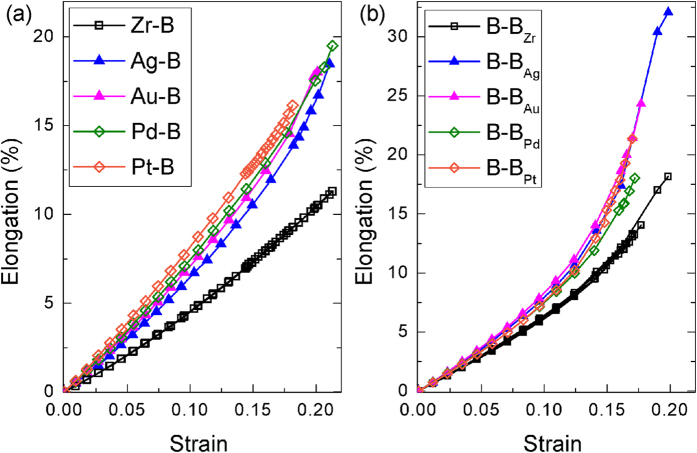
(**a**) Elongation-strain curves of X-B bonds and Zr-B bonds in (Zr, X)B_2_ during the basal plane shear. (**b**) Elongation-strain curves of B-B_X_ bonds and B-B_Zr_ bonds in (Zr, X)B_2_ during the prismatic plane shear. Here, B-B_X_ bond means the B-B bond surrounding X element. Elongation-strain curves of Zr-B bond and B-B_Zr_ bond obtained from different simulations are almost coincident with each other. Therefore, they are not distinguished by different symbols.

**Table 1:** Valence state and electronegativity (Pauling’s scale) of element Zr, W, Ag, Au, Pd and Pt, and their charges when dissolving into ZrB_2_, shear modulus (*G*) and ideal shear strength (*τ*) of ZrB_2_ with different alloying elements and their relative change with respect to those of ZrB_2_.

		Zr	W	Ag	Au	Pd	Pt
Valence state		4d^2^5s^2^	5d^4^6s^2^	4d^10^5s^1^	5d^10^6s^1^	4d^10^	5d^9^6s^1^
Electronegativity		1.33	2.36	1.93	2.54	2.20	2.28
Charge		+1.15	+0.46	+0.70	+0.42	+0.27	+0.39
basal plane shear	*G*_*b*_ (GPa)	252.2	255.9	233.2	225.4	228.9	220.6
	*G*_*b*_/*G*_*b*0_−1 (%)	—	+1.5	−7.5	−10.6	−9.2	−12.5
	*τ*_*b*_ (GPa)	41.8	39.7	35.2	33.4	35.1	32.2
	*τ*_*b*_/*τ*_*b*0_−1 (%)	—	−5.0	−15.8	−20.1	−16.0	−23.0
prismatic plane shear	*G*_*p*_ (GPa)	244.1	256.3	243.4	238.1	240.1	236.5
	*G*_*p*_/*G*_*p*0_−1 (%)	—	+5.0	−0.3	−2.5	−1.6	−3.2
	*τ*_*p*_ (GPa)	37.1	36.1	35.1	34.2	34.9	32.9
	*τ*_*p*_/*τ*_*p*0_−1 (%)	—	−2.7	−5.4	−7.8	−5.9	−11.3

Subscript b and p respectively means the basal plane shear and the prismatic plane shear.
