# Supplementary material for: Reducing the Ideal Shear Strengths of ZrB2 by High Efficient Alloying Elements (Ag, Au, Pd and Pt)
Source: Sci Rep. 2017 Feb 24;7:43416. doi: 10.1038/srep43416 (PMC5324072; doi:10.1038/srep43416)
Supplement: Supplementary Information [file srep43416-s1.doc]

**Supplementary Material of “Reducing the Ideal Shear Strengths of ZrB_2_ by High Efficient Alloying Elements (Ag, Au, Pd and Pt)”**

Fu-Zhi Dai and Yanchun Zhou[[1]](#footnote-2)

Science and Technology of Advanced Functional Composite Laboratory, Aerospace Research Institute of Materials & Processing Technology, Beijing 100076, China

**Detailed Analyses on Chemical Bonds of ZrB_2_**

Figure S1 illustrates the band structure of ZrB_2_ and the electron density maps on (1120) plane of different orbits. In the figure, the correlation between an orbit and its electron density map is highlighted by colors. It is clear that electrons occupying the three lowest energy bands all display *σ* bond like distribution with high electron density between B atoms. For the band with the fourth lowest energy level (the fourth band for simplicity), electrons concentrate around each B atom instead of aggregating between B atoms, which represents π bond like distribution. The analyses reveal that the three lowest energy bands are almost contributed by B-B *σ* bonds, while π bond electrons mainly occupy energy bands higher than the third band. Then, the lowest energy of the fourth band (-4.1eV) can be taken as a rough lower bond of the energy of π bond electrons. In addition, the characteristics of electron distribution of the valence band (the fifth band) include π bond like distribution and hybridization of B-2p orbit and Zr-4d orbit, which contributes to the strong bonding between B and Zr. Similar analyses on chemical bonds have also been reported in reference [24].


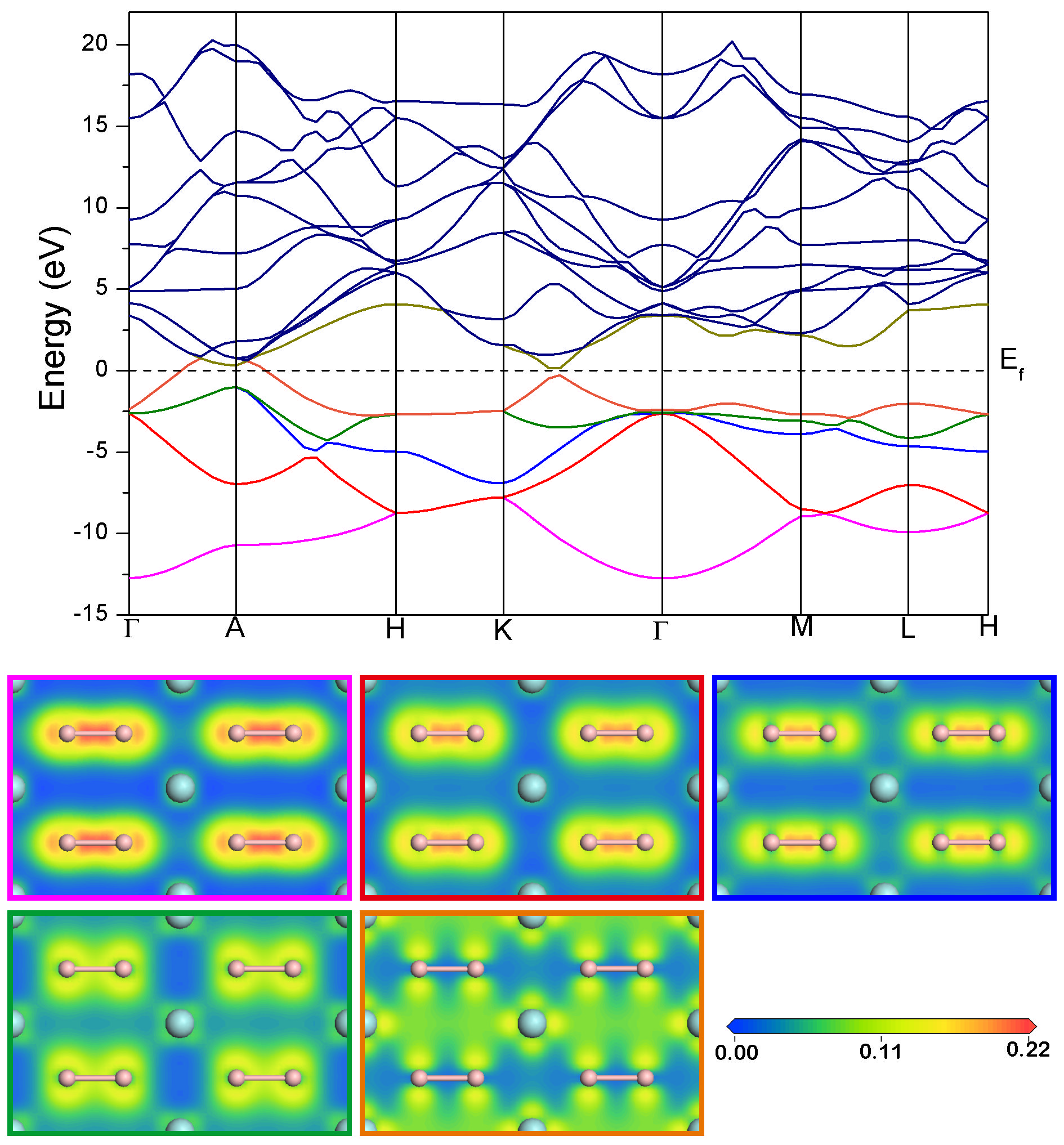


Figure S1. Band structure of ZrB_2_ and decomposed electron density maps on the (1120) plane of different orbits. The correlation between an orbit and its electron density map is highlighted by colors.

1. Corresponding author. Tel.: +86 10 88524221; fax: +86 10 68759874.

   *E-mail addresses:* yczhou@imr.ac.cn (Y. Zhou). [↑](#footnote-ref-2)
